# Dr. Edwin C. Baxter

**Published:** 1895-10

**Authors:** 


### Obituary.

### DR. EDWIN C. BAXTER.

Died July 14, 1895, at Albany, New York, of typhoid fever, Dr. Edwin C. Baxter.

The announcement of the departure of young and old is always accompanied with a feeling of sadness that sooner or later all must pass over into the silent land whether their lives have been useful or otherwise.

The dental profession has suffered serious loss the past few years in the deaths of many of its brightest men, and among these can certainly be classed the friend of many years, Dr. Baxter, of Albany. His ability in his profession was never questioned; as an operator he had few, if any, superiors. The basis of his professional excellence was laid when mechanical and operative dentistry were considered of primal importance. To this practical education was superadded his collegiate work in the Pennsylvania College of Dental Surgery of Philadelphia, from which he graduated in 1866, leaving nothing in his training to be desired.

His natural bent seemed to the writer to be away from the stirring activities of professional life, hence he was rarely met with outside of his own State in conventions; but in it he was regarded with the respect due to a man of eminent worth and solid judgment. He was one of the examining board of New York State, and had much to do with the passage of the recently enacted law placing dentistry under the State Board of Regents.

The family of the Baxters were originally from Maine, of English descent.

He began the study of dentistry with Dr. Edwin Parsons, of Portland, Maine. From there he went to Philadelphia and graduated, subsequently becoming the assistant and, at a later period, the partner of Professor C. N. Peirce. He settled in Albany about twenty-five years ago.

Dr. Baxter was married in 1873 to Lydia Ryerson Sprague, of Long Island, who survives him.

Thus passed from life one of the warmest friends and the gentlest of men; but his life, kind deeds to those who needed help, and his faithful work will be a lasting memory to those who knew him best.

